# Obstructive sleep apnea and diabetes

**DOI:** 10.1111/1753-0407.13494

**Published:** 2023-11-05

**Authors:** Zachary Bloomgarden

**Affiliations:** ^1^ Department of Medicine, Division of Endocrinology, Diabetes and Bone Disease Icahn School of Medicine at Mount Sinai New York New York USA

Obstructive sleep apnea (OSA) is a sleep‐related breathing disorder, in which relaxation of supportive structures of the throat during sleep leads to collapse of the retropharyngeal soft tissue, blocking the upper airway. An early recognition of OSA is Charles Dickens' description of a “wonderfully fat boy” in *The Pickwick Papers*, published in 1836, noting the boy's excessive appetite and many attacks of sleep. Although in the past OSA prevalence was estimated as being 2%–4% among adults,^1^ more recent studies have suggested mild and more severe OSA to be present in 26% and 12% of adults, amounting to >900 million and >400 million persons worldwide, respectively,^2^ and a current meta‐analysis of 99 studies of >47 000 persons reported adult prevalence of OSA of 56% and of moderate to severe OSA of 37%.^3^ In this study, 87% of truck drivers, 78% of bus drivers, and 69% of “elderly” persons had OSA, with obesity (particularly of the neck and abdomen), male sex, hypertension, alcohol, and cigarette use all associated with greater risk.^3^ OSA occurs more commonly among persons with diabetes, and is associated with insulin resistance, polycythemia, hypercoagulability, nonalcoholic fatty liver disease, and cardiovascular disease (CVD), including heart failure, with arrhythmias, and with increased mortality.

OSA can be characterized during polysomnography by the documentation of apnea, defined as ≥90% reduction in airflow for >10 s and/or by hypopnea, ≥30% reduction in airflow for >10 s associated with >3% reduction in arterial oxygen saturation or by arousal from sleep. The accepted criteria for OSA severity rank it according to the apnea‐hypopnea index (AHI) as mild, 5–15 events/h, moderate, >15–30 events/h, and severe, >30 events/h. A sense of the typical findings in persons with OSA can be obtained from currently used screening instruments. STOP‐Bang gives 1 point each for snoring, tiredness, observed apnea, and the presence of hypertension, body mass index (BMI) >35 kg/m^2^, age >50 years, neck circumference >40 cm, and male gender with the presence of ≥3 of the criteria considered positive, and NoSAS uses a related set of criteria, adding 4 points for neck circumference ≥40 cm, 3 points for BMI 25–29.9 and 5 points for BMI≥30, 2 points for a history of snoring, 4 points for age > 55 years, and 3 points for male sex, with a total of ≥8 being considered a positive score.^4^ In a study of 201 persons undergoing polysomnography, of whom roughly one quarter each had mild, moderate, and severe OSA or did not have OSA, both of the screening measures had modest predictive power for OSA, with NoSAS≥8 having somewhat higher C‐statistic (area under the sensitivity vs 1‐specificity curve) than STOP‐Bang≥3, and the Epworth sleepiness scale (ESS) of self‐assessed likelihood of falling asleep in eight scenarios had a C‐statistic of approximately 0.5 in predicting OSA, suggesting it to be little better than a coin‐flip in determining whether an individual had OSA.^4^

Diabetes appears to be strongly related to OSA. A systematic review of 41 studies of adults with diabetes showed 60% OSA prevalence.^5^ In a combined analysis of 146 519 participants in the Nurses' Health Study and Health Professionals Follow‐up Study, 13.8% of men and 6.4% of women report being diagnosed as having OSA; diabetes was associated with a 2.14‐fold greater likelihood of OSA, although much of this was explained by the association both of diabetes and of OSA with greater weight and waist circumference.^6^ A meta‐analysis of 22 studies of persons with type 1 diabetes showed an OSA prevalence among adults of 52%.^7^ In a UK primary care records database including 360 250 persons with type 2 diabetes (T2D) vs 1 296 489 not having diabetes, OSA was identified by diagnosis code in 3110 (0.88%) with diabetes and in 5968 (0.46%) nondiabetic persons; although this likely shows a marked degree of underdiagnosis, there was a 1.76‐fold greater rate among persons with diabetes, with OSA 2.0‐fold more likely with BMI 25–30 and 8.3‐fold more likely with BMI > 30.^8^ Conversely, OSA is associated with greater risk of diabetes. A meta‐analysis of 16 cohort studies with 338 912 participants of whom 19 355 developed T2D over a median 10.5 year follow‐up, the relative risk of developing diabetes was 1.4‐fold among persons with OSA.^9^ In a subsequent metaanalysis of 25 studies with 154 948 participants, the odds ratio of developing diabetes among persons with OSA was 2.15 in cohort studies, and 3.62 in cross‐sectional studies, the risk increasing with greater OSA severity.^10^ Among 9076 persons studied in the National Health and Nutrition Examination Survey (NHANES) from 2005 to 2008, self‐reported OSA was associated with an odds ratio for diabetes development of 1.46.^11^ Newer measures of OSA severity may lead to better appreciation of the relationship between OSA severity and diabetes, with a recent study suggesting the AHI to be less sensitive than a sleep breathing impairment index in predicting the likelihood of diabetes.^12^

A number of sleep characteristics are related to diabetes. Among 6289 persons evaluated in NHANES from 2015 to 2018, excessive daytime sleepiness (EDS) was noted in 31% of the 895 persons with diabetes but in 26% of nondiabetic persons; with EDS associated with younger age, lower income, thyroid disease, depression, sleep apnea, inadequate sleep, higher BMI, hypertension, smoking, less physical activity, and worse diet, although also with greater access to health care, white race, and higher education level.^13^ In a study of 1841 persons with hypertension and OSA, at 7‐year follow‐up diabetes developed in approximately 13% of those with nondipping hypertension at baseline (nocturnal mean blood pressure decreasing <10% compared to daytime levels) and in approximately 8% of those with a nocturnal “dipping” pattern.^14^ Another study, of 1478 persons with hypertension and OSA, found that for each SD increase in midnight cortisol level there was a 26% increase in the likelihood of development of diabetes, with a significant effect seen only among persons with moderate or severe OSA.^15^ Among 13 346 persons with T2D in the UK Biobank Project, high OSA risk was based on a history of observed snoring, “often” or “always” experiencing daytime sleepiness, and either having BMI≥30 or hypertension; those at high risk had a mean glycated hemoglobin (HbA1c) of 7.27, whereas those at low OSA risk had significantly lower mean HbA1c of 7.17%; in that study both long (≥9 h) and short (≤6 h) sleep duration were associated with 0.1% higher HbA1c levels than that of those sleeping an average of 7–8 h.^16^

OSA is associated with increase in CVD, intermittent hypoxemia and hypercarbia leading to hypertension, atherosclerosis, heart failure, and the development of heart rhythm disturbances^17^, ^18^; all these complications correlated with OSA severity in a study of 1514 persons with OSA.^19^ Among 1022 persons with studied with polysomnography and followed over a mean 3‐year period, those with AHI 4–36 had 1.75‐fold greater and those with AHI > 36 had 3.3‐fold greater likelihood of stroke or death that those with AHI ≤ 3.^20^ In a study of 2031 persons with hypertension and OSA, those with greater levels of insulin resistance were at greatest risk both of coronary heart disease and of stroke.^11^ There may be better measures of OSA severity than the AHI, with new measures of ventilatory deficit, hypoxic burden, and arousal characteristics appearing to be more sensitive than the AHI in their association with CVD and mortality.^21^, ^22^

A number of approaches are beneficial in the treatment of OSA. Intensive lifestyle intervention in persons with OSA and obesity is associated with both weight loss and sustained reduction in the AHI, with resolution of OSA in many persons.^23^ Similarly, a randomized controlled trial of the combination of pherntermine with topiramate in obese persons with OSA showed weight loss in association with reduction in AHI by 31.5 vs. 16.6 events/hour.^24^, ^25^ A meta‐analysis of 32 studies with 2310 patients undergoing bariatric surgery showed a decrease in BMI from 43.5 to 32.1, a reduction in AHI from 35 to 14.2, and remission of OSA in 64%.^26^ In a 3‐month study, liraglutide was associated with reduction in AHI along with weight loss,^27^ and the use of empagliflozin and of ertugliflozin in persons with diabetes was associated with reduction in risk of new development of OSA by 52% and 48%, respectively.^28^ Continuous positive airway pressure (CPAP) treatment is effective for OSA and leads to marked improvement in daytime sleepiness as measured by the ESS and is also associated with modest reduction in blood pressure.^29^ There is, however, no evidence that CPAP has CVD outcome benefit, with meta‐analysis of 18 studies of 4146 patients with OSA showing no significant effect on total CVD events, on stroke, or on mortality (Table 1).^29^

**TABLE 1:** Effect of CPAP on cardiovascular outcomes: meta‐analysis of 18 studies of 4146 patients with OSA, from reference.

	OR (95% CI)	*p* value
CV events	0.84 (0.62 to 1.13)	.25
Mortality	0.85 (0.35 to 2.06)	.72
Stroke	0.56 (0.18 to 1.73)	.32
Epworth sleepiness score	−1.78 (−2.31 to 1.24)	.000001
Systolic BP	−2.03 (−3.64 to −0.42)	.01

Abbreviations: CI, confidence interval; CPAP, continuous positive airway pressure; OR, odds ratio; OSA, obstructive sleep apnea.

OSA is, then, an important condition leading to numerous complications affecting patients with diabetes.^30^ OSA is of particular concern in its strong association with the development of diabetes and with a large variety of CVD outcomes, with the severity of OSA correlating with the likelihood of such outcomes. Although standard treatment with CPAP improves the primary OSA symptom of excessive daytime sleepiness, and has some blood pressure reducing benefit, it is not clear that CPAP reduces development of diabetes or of CV complications. New approaches to OSA assessment and treatment are eagerly awaited.
